# Supplementary material for: Splice-Junction-Based Mapping of Alternative Isoforms in the Human Proteome
Source: Cell Rep. Author manuscript; Available in PMC 2020 Jan 15. (PMC6961840; doi:10.1016/j.celrep.2019.11.026)
Supplement: 3 [file NIHMS1546469-supplement-3.zip › DF2/PXD0010154/AdrenalGland-123-Q9C037-ESQEHQTHAMAPIDEAFESYREK.pdf]

A

Predicted sequence disorder and sequence features of Q9C037

Peptide: ESQEHQTHAMAPIDEAFESYREK Junction: sp|Q9C037|TRIM4_HUMAN|ENSG00000146833|SE2|43831|chr7|99909660|99916779|−0|r84|T1 TrNovel: FALSE

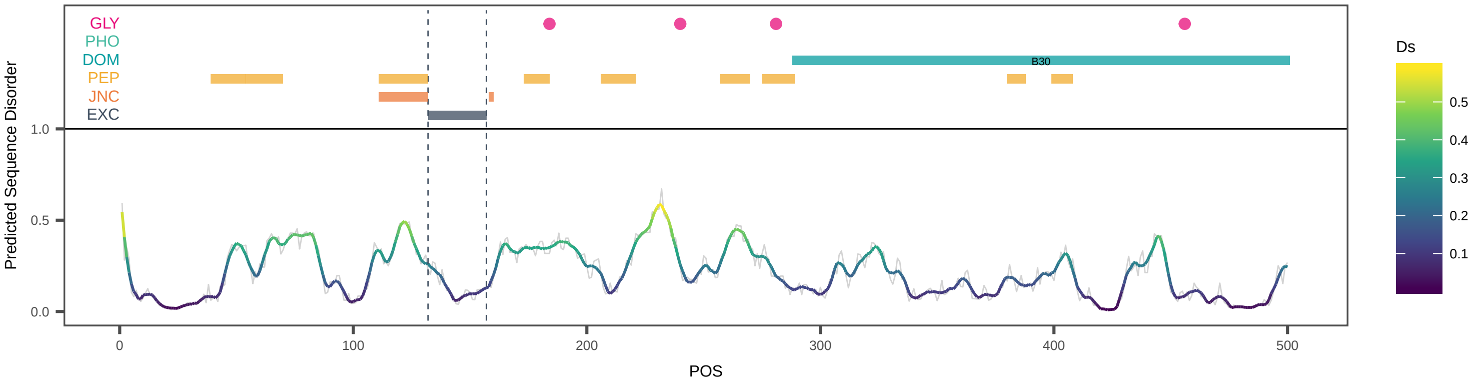

B

Distribution of sequence disorder in excised
vs. mapped and non−excised regions of protein

M−W P−value vs. mapped: 8.8e−10 vs. non−excised: 0.00263

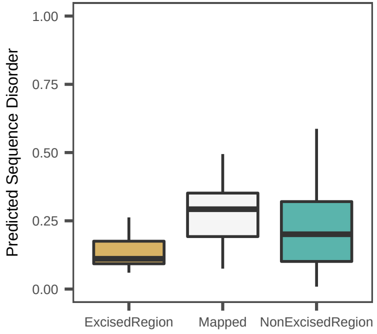

C
